# 736. Impact of COVID-19 on Mortality Associated with *Pseudomonas aeruginosa* Bloodstream Infection in the Veterans Health Administration System

**DOI:** 10.1093/ofid/ofac492.027

**Published:** 2022-12-15

**Authors:** Leila S Hojat, Brigid Wilson, Federico Perez, Robert A Bonomo

**Affiliations:** Case Western Reserve University/ University Hospitals Cleveland Medical Center, Cleveland, OH; Louis Stokes Cleveland VA Medical Center, Cleveland, Ohio; Louis Stokes Cleveland VA Medical Center, Cleveland, Ohio; Case Western Reserve University/ Louis Stokes Cleveland VA Medical Center, Cleveland, OH

## Abstract

**Background:**

*Pseudomonas aeruginosa* bloodstream infection (PA-BSI) is associated with high mortality. The extent to which the COVID-19 pandemic has introduced challenges to diagnosis and treatment of this infection is unknown. In this study, we examined the direct and indirect effects of COVID-19 on PA-BSI associated mortality in the Veterans Health Administration (VHA) population.

**Methods:**

We identified patients within the VHA database with PA-BSI identified between January 1, 2009 and December 31, 2019 (pre-COVID) and January 1, 2020 to December 31, 2021 (COVID). Extracted data included age, race, ethnicity, Charlson Comorbidity Index (CCI), susceptibility testing, treatment, and COVID-19 infection. Antimicrobial resistance was characterized as pan-susceptible, any unclassified resistance, multi-drug resistant (MDR), difficult to treat resistant (DTR), and extremely or pan-drug resistant (XDR/PDR). Active therapy was defined as an effective antibiotic initiated within 48 hours of blood culture collection. We used logistic regression to assess the impact of each factor on 30-day mortality.

**Results:**

We identified 7,578 patients with PA-BSI and known 30-day mortality status, which was 24% overall (Table 1). Age and CCI were higher during COVID, while effective treatment and resistance improved (Table 2). In the multivariate analysis, concomitant COVID infection tripled the odds of mortality (odds ratio [OR] 3.00, 95% confidence interval [CI] 1.40–6.37) (Figure 1). However, the COVID period itself was not associated with higher mortality. Effective treatment was associated with 19% lower odds of mortality (OR 0.81, 95% CI 0.66–1.01), while DTR and XDR/PDR nearly doubled mortality (OR 1.75, 95% CI 1.23–2.47 and OR 2.06, 95% CI 1.25–3.34, respectively).

**Conclusion:**

We observed higher odds of mortality in patients with PA-BSI and COVID-19 coinfection, supporting the hypothesis that COVID-19 directly impacts PA-BSI outcomes. Annual PA-BSI incidence and associated 30-day mortality, however, were similar during the COVID period. Favorable resistance trends, effective treatment, and a low rate of PA-BSI and COVID-19 coinfection may explain these findings, despite increased age and comorbidities in this vulnerable population.

**Disclosures:**

**Federico Perez, MD, MS**, Accelerate: Grant/Research Support|Merck: Grant/Research Support|Pfizer: Grant/Research Support **Robert A. Bonomo, MD**, Merck: Grant/Research Support|Pfizer: Honoraria|VenatoRx: Grant/Research Support|Wockhardt: Grant/Research Support.

